# Limb Salvage for Musculoskeletal Tumors in the Austere Environment: Review of the Literature With Illustrative Cases Regarding Considerations and Pitfalls

**DOI:** 10.5435/JAAOSGlobal-D-19-00172

**Published:** 2020-10-01

**Authors:** S. Craig Morris, Scott C. Nelson, Lee M. Zuckerman

**Affiliations:** From the Department of Orthopaedic Surgery, Loma Linda University, Loma Linda, CA (Dr. Morris, Dr. Nelson); the Department of Surgery, Hopital Adventiste d’Haiti, Port-au-Prince, Haiti (Dr. Nelson); and the Division of Orthopaedic Surgery, Department of Surgery, City of Hope National Medical Center, Duarte CA (Dr. Zuckerman).

## Abstract

Although there is literature discussing the treatment of acute and chronic trauma in austere environments, no literature or guidelines for the treatment of musculoskeletal tumors exist. This series discusses case examples with considerations and pitfalls of performing limb-salvage surgery in an underserved location.

Cases of limb-salvage surgery performed by the same orthopaedic oncologist in Haiti and the Dominican Republic are discussed with a review of the literature on limb salvage for musculoskeletal tumors in developing nations.

All patients successfully underwent limb-salvage surgery after considering multiple factors including tumor type and location. Patients with metastatic disease, likelihood of substantial blood loss, and poor health were not candidates for limb-salvage surgery.

Medical missions and the development of partnerships with established training programs make limb salvage a greater possibility. Knowledge of the facility, anesthesia support, and instrumentation available is vital. Advanced imaging, blood products, and allograft are likely unavailable or difficult to obtain. Established continuity of care is necessary, and training of the local surgeon should be provided. Surgery should only be considered if it is safe and provides more of a benefit to the patient than an amputation.

There exists a tremendous global problem regarding access to surgical care, with a model estimating that 4.8 billion people lack access to surgery.^1^ Not surprisingly, certain regions have greater difficulty accessing surgery and bear a greater burden of disease.^1,2,3,4,5^ Because of this need, the World Health Organization has become involved in improving access to, and quality of, surgical care in low-income and middle-income countries.^6^ The need for and strategies to address global orthopaedic trauma care have been described.^4,7,8,9,10^ Although musculoskeletal tumors are rare, there still exists a great need for surgical care of these conditions. Barriers to surgical care in low-income and middle-income countries include structural aspects of health care, cultural beliefs and attitudes, and financial barriers.^11^ Improving trauma care in these countries has been projected to result in substantial health and economic benefits.^12^ There exists an opportunity for obtaining similar benefits through improvement in the care of musculoskeletal tumors. Discussion of limb-salvage surgery in the austere environment is limited to the trauma literature.^13,14,15,16^ When resources are not limited, limb salvage is the standard for extremity tumor resection when adequate function can be maintained.^17^ In areas where resources are limited and care may be delayed, limb salvage may not always be possible. This article provides an organized, comprehensive discussion of the considerations necessary to improve the care of musculoskeletal tumors in underserved regions of the world, including the evaluation of facilities and resources, patient-specific factors, and long-term care capabilities that should guide treatment decisions. Illustrative cases are included to discuss situations when limb salvage may be possible and the obstacles that may be encountered. The use of patient data presented in this article was approved by our institutional review board. It must be emphasized that these are general considerations that must be adapted as appropriate to the challenges and resources unique to each location and circumstance. Table 1 lists questions and considerations that should be applied before embarking on complex limb-salvage surgery in the austere environment.

**Table 1:** Questions and Considerations to Evaluate the Viability of Performing the Limb-Salvage Surgery

Yes Required for Complex Limb-Salvage Surgery	Considerations
Has the surgeon operated there previously?	Is the country/government stable?
Is the surgeon able to direct postoperative care or return to the country if needed?	Can the pathology be evaluated?
Has the anesthesiologist worked there previously?	Is there a physical therapist?
Is there a local surgeon that can manage complications and follow the patient long term?	Is there a prosthetist/material to make prosthetics?
Will training of a local surgeon be provided?	Is the patient able to pay for testing, blood, or other costs that may not be needed with another surgery?
Is the required equipment available and working?	Is a local blood bank available?
If specialized equipment is needed, can it be brought into the country?	
Can the operating room handle a power outage?	
Is the available imaging adequate to perform the proposed surgery?	
Does the patient have localized disease?	
Will the patient survive without other treatment modalities if they are not available?	
Is the patient able to tolerate the expected blood loss?	
Is the expected functional outcome better than an amputation?	
Is the patient able to follow-up in-person and can be contacted by phone?	
Will outcomes be evaluated and improvements in care be provided?	

## Facilities and Equipment

All cases were completed by one orthopaedic oncologist at the Haiti Adventist Hospital in Haiti and the CURE Hospital in the Dominican Republic. The surgeon had made prior trips to Haiti and performed multiple surgeries for osteochondromas, fractures, and small soft-tissue tumors before attempting the surgeries described. The surgeries were performed with local anesthesiologists, a local and international full-time orthopaedic surgeon, and local operating room staff and nurses. Long-term care was provided by the full-time orthopaedic surgeons. Both hospitals routinely performed surgery for acute and chronic trauma, chronic infections, and limb deformity in adult and pediatric patients. The hospital in Haiti was in an enclosed compound where the surgeon stayed with armed guards at the gate. Cell phone reception and Wi-Fi were intermittently available.

For reconstruction, small and large fragment sets, intramedullary nails, external fixators, a large C-arm, dermatome, multiple types of suture, pneumatic tourniquets, and drills and saws were available on-site. Periarticular plates and joint replacements were not routinely available. A standard radiograph machine and ultrasonography, but no portable radiographs or advanced imaging, were available. The donated equipment was brought into Haiti by volunteers because shipments were unlikely to arrive at the hospital. Inspection at the airport was common, but the equipment was usually released by paying a fee. Back-up power generators for the hospital were available as well as a physical therapist and cast technician. Cloth gowns and drapes, lap pads, cautery equipment, tourniquets, and external fixators were cleaned and sterilized for multiple uses. Blood was obtained by directed donor or on a limited basis through the Red Cross. Chemotherapy, radiation, allograft, and prosthetics for amputees were not available. All pathology discussed was transported to the United States for review.

## Diagnosis: Imaging

**Figure 1:**
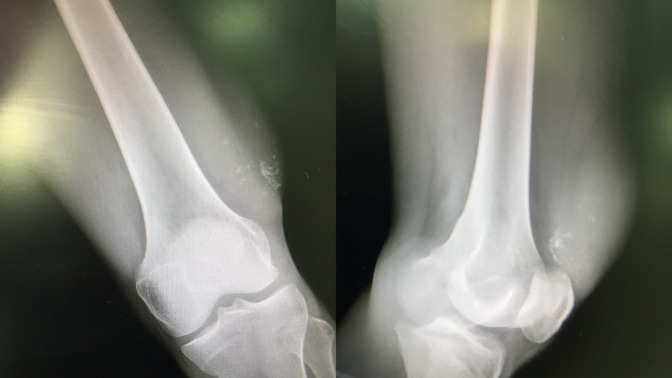
AP and oblique radiographs of the distal femur showing a soft-tissue mass near the knee joint with calcifications. These characteristic findings lead to synovial sarcoma being in the differential of possible lesions. The mass was mobile and away from the neurovascular structures and a chest radiograph was normal. Therefore, limb-salvage surgery could be considered.

**Figure 2:**
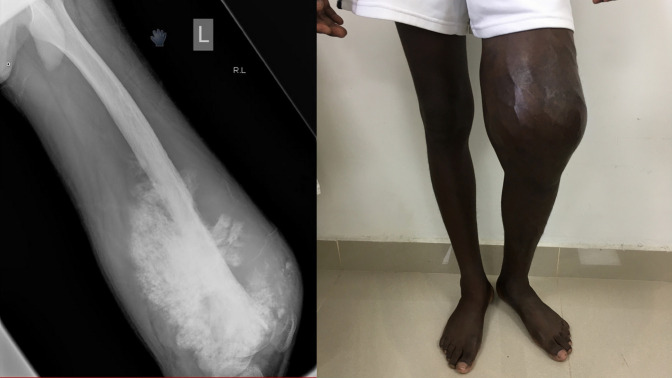
Lateral radiograph of the left femur showing a lesion of the distal femur with bone formation and a sun burst pattern characteristic of osteosarcoma. Clinical photograph demonstrating associated swelling and deformity about the distal femur. Limb salvage was not chosen due to the risk of the surgery, lack of chemotherapy, and lung nodules on chest radiographs.

The value of radiographs along with a history and physical to obtain a diagnosis should not be underestimated. Although a definitive diagnosis may not be possible, radiographs can guide treatment. In cases of soft-tissue tumors, calcifications, underlying bone erosions, or phleboliths may lead to a diagnosis.^18,19,20^ A well-defined or lobulated juxta-articular soft-tissue mass with calcifications is suggestive of synovial sarcoma^19^ (Figure 1). In cases of osteosarcoma, bone formation, cortical destruction, and periosteal reaction are typical^20^ (Figure 2). Some centers may have advanced imaging modalities, but the patient may have to pay for these services and traveling to these centers may not be possible. Although these tests add information, they may not be necessary for treatment. One study noted a 32.4% prevalence of inappropriate advanced imaging ordered before referral for musculoskeletal tumors when resources were not limited.^21^ For the surgeon who does not specialize in oncology, consultation with a musculoskeletal radiologist or orthopaedic oncologist is a vital link in making a diagnosis or deciding whether a biopsy is necessary. An image of the radiograph sent by e-mail can be accomplished in many circumstances. Benign lesions, including hemangiomas or osteochondromas, can be identified with radiographs, and consultation can prevent unnecessary procedures. If the diagnosis can be obtained by radiograph alone, then the surgeon who does not specialize in oncology, but has experience treating the specific tumor, can proceed with an appropriate treatment plan with confidence.

## Diagnosis: Biopsy

For any tumor that might require a biopsy, consultation with an orthopaedic oncologist is vital to the treatment and neither a biopsy nor definitive treatment should be attempted by an inexperienced surgeon. A biopsy should always be done under the guidance of a surgeon who performs limb-salvage surgery and ideally by the operating surgeon.^22^ Image-guided biopsies may not be possible, and an inappropriate biopsy can necessitate amputation.^23^ Biopsy principles and techniques can be taught to the local surgeon during initial trips to the region. A pathologist may not be available, and the sample may need to be transported out of the country. If the tumor is aggressive enough on radiographs to warrant a wide resection, then the treatment could be considered without a prior diagnosis, but this is a decision that should be made by the surgeon performing the definitive surgery.

## Case Application

**Figure 3:**
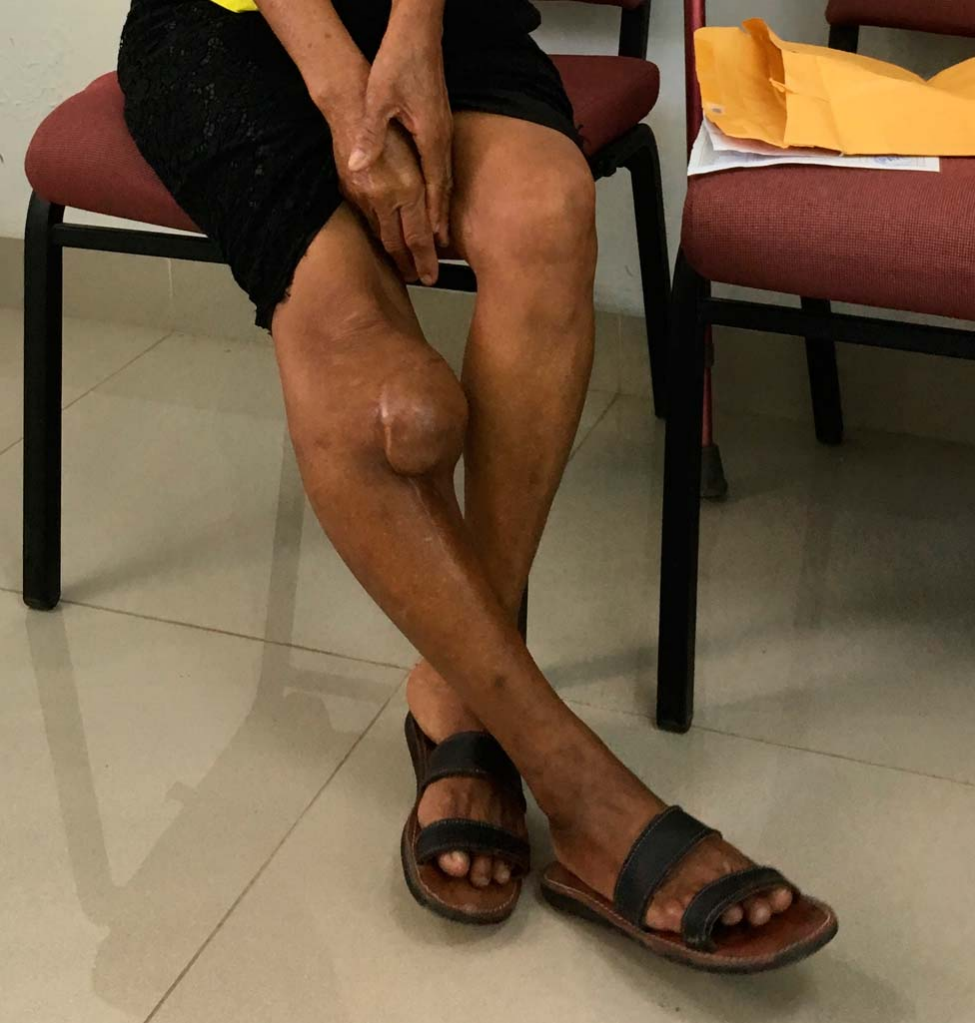
Clinical photograph demonstrating a large mass at the right proximal tibia with a varus deformity.

**Figure 4:**
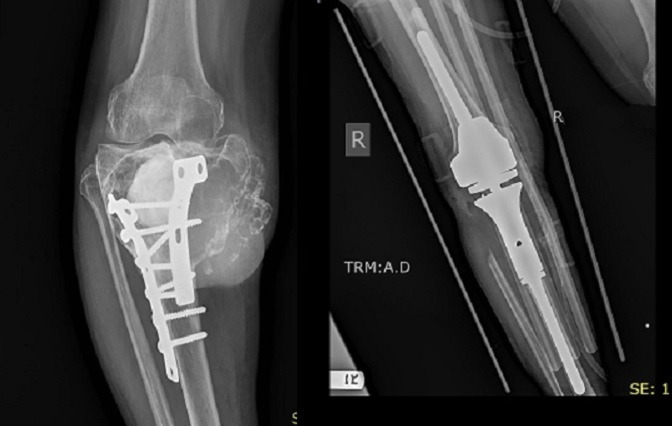
AP radiograph of the right knee preoperatively with recurrent giant cell tumor that was found to have undergone sarcomatous transformation. AP radiograph of the right knee after resection and reconstruction with a proximal tibial replacement.

A 57-year-old woman presented in Haiti with a right proximal tibia lesion (Figure 3). Two years prior to presentation, she underwent a curettage resection and pathology was consistent with a giant cell tumor (GCT) of bone. The tumor had recurred, causing severe pain, deformity, and difficulty with ambulation. The patient underwent an en bloc resection with reconstruction using a donated proximal tibia replacement, medial gastrocnemius flap, and split-thickness skin graft (Figure 4). The decision was made to not perform a biopsy by the orthopaedic oncologist because it would not change management. The severe destruction of the joint necessitated complete resection. Therefore, surgical resection with a margin of normal tissue was chosen to accommodate for both the diagnosis of a GCT and a malignancy. If the tumor was amenable to an intralesional resection, a biopsy would have been performed. The final pathology demonstrated malignant change of the GCT into a sarcoma with negative margins, and therefore, the treatment was appropriate. If the diagnosis of a standard GCT was assumed and an intralesional resection was performed, the surgery would have been inappropriate and required a second, more extensive surgery, or an amputation. The patient's postoperative course was without incident. At the 2-year follow-up, the patient is disease- and pain-free, is able to walk without assistance, and has active knee motion from 0 to 120°.

For the diagnosis of a sarcoma, the patient should be monitored for both local recurrence and metastatic spread. She has had scheduled visits every 3 months for the first 2 years to examine the area, and to obtain new radiographs of the chest, knee, and tibia. Subsequently, she will have visits with radiographs every 6 months until year 5 and then yearly visits thereafter. The specific schedule of follow-up should be catered to both the type of tumor resected and the reconstruction performed. These factors should be considered regarding whether the patient can afford to travel for follow-up and any costs that may be required for the visit and imaging.

In addition to being followed for local recurrence and metastatic spread, the patient will have to be monitored for implant failure. If this occurs, this patient will likely have to undergo an above-the-knee amputation as opposed to a through-the-knee amputation that could have been a primary operation. It should not be assumed that the implant can be revised and the choice for limb salvage in this case becomes a multifactorial choice regarding the risks of implant failure with the benefits of limb salvage versus the functional outcome a primary amputation would provide. The availability of prosthetics for amputees in the region must be considered, along with the patient's age, living situation, functional status, prognosis, and ability of the local surgeon to either remove the implant or perform an amputation above the implant stem if necessary.

## Chemotherapy and Radiation

**Figure 5:**
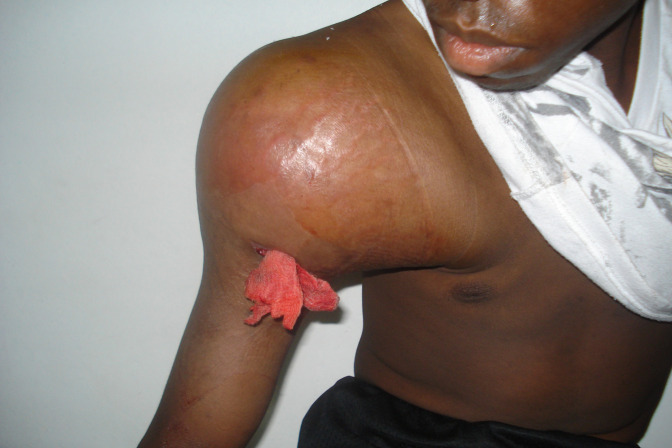
Clinical photograph of a 19-year-old man with a chronic draining wound of the right shoulder and severe pain. Radiographs of the shoulder demonstrated findings most consistent with an osteosarcoma. Given the lack of chemotherapeutic options, the patient's prognosis is poor. Owing to the risk of infection and blood loss with surgical reconstruction, palliative amputation would be an option for both resection of the tumor and pain control.

One pitfall regarding this patient is the inability to administer chemotherapy. For high-grade osteosarcomas and primary bone sarcomas, chemotherapy is standard, and without chemotherapy, there is only a 20% 5-year survival rate for osteosarcoma.^24,25^ Limb salvage for most bone sarcomas without chemotherapy is unlikely to result in long-term survival. The hope for this patient is that micrometastases are not present and the sarcoma was identified in the early stages of transformation from a benign GCT. Ideally, this patient will have an improved quality of life and functional status if she does develop metastatic disease. Similarly, many soft-tissue sarcomas require radiation therapy to prevent local recurrence.^26,27^ Tumors that require chemotherapy or radiation should be approached with caution because limb salvage may be a more extensive procedure with more complications for a patient that may not survive to appreciate the benefits. At minimum, a chest radiograph should be performed to evaluate for lung metastases because metastatic disease found on presentation is likely to result in a shorter course of survival. The complexity of the surgery should also be a consideration. A superficial soft-tissue sarcoma where a 2-cm margin of normal tissue can be resected safely with primary closure or a skin graft is a better candidate than a tumor that is compressing the major vasculature or requires a free flap for coverage. In many cases, palliative care or a palliative amputation should be the treatment of choice if limb-salvage surgery is risky or extensive (Figure 5).

## Treatment Goals

The ultimate goal of the treatment should be to provide the patient with the best functional outcome and highest prognosis for survival while minimizing complications. Owing to this, an understanding of the biology of the tumor and goals of surgery are necessary.

## Case Application

**Figure 6:**
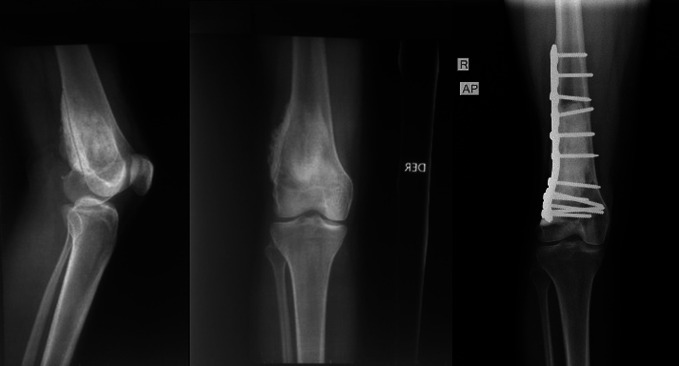
AP and lateral radiographs of the right knee showing a distal femur parosteal osteosarcoma. AP radiograph of the right knee after resection and reconstruction.

A 21-year-old woman presented in the Dominican Republic after an intralesional excision of a right distal thigh mass 7 years ago. The pathology at that time was felt to be consistent with heterotopic ossification. She developed increasing pain and was found to have a mass arising from her prior resection site with imaging concerning for a parosteal osteosarcoma. A biopsy was performed under the direction of the orthopaedic oncologist and was consistent with a low-grade osteosarcoma. In this case, proper resection of the tumor could result in a cure because chemotherapy and radiation are not standard. The patient underwent resection of the tumor, including resection of the biopsy tract, and reconstruction with a plate and cement (Figure 6). The final pathology was consistent with a parosteal osteosarcoma with negative margins. Four years after surgery, the patient is disease free and without pain or functional deficit.

An understanding of the natural progression of the tumor is vital in this case because limb-salvage surgery is possible with minimal reconstruction and a good overall prognosis. These decisions are not just limited to malignant tumors as benign tumors can progress to the point of requiring a determination about amputation versus limb-salvage surgery. In areas with poor access to health care, benign and benign aggressive tumors are more likely to have advanced in severity before presentation. This case again illustrates the importance of consultation with the operating surgeon, as resection of the biopsy tract is needed to prevent local recurrence, and a poorly performed biopsy can result in the need for amputation.^28,29^ If the biopsy contaminated the popliteal vessels, limb salvage would not have been possible as the risk of performing a vascular reconstruction was unacceptable.

## Case Application

**Figure 7:**
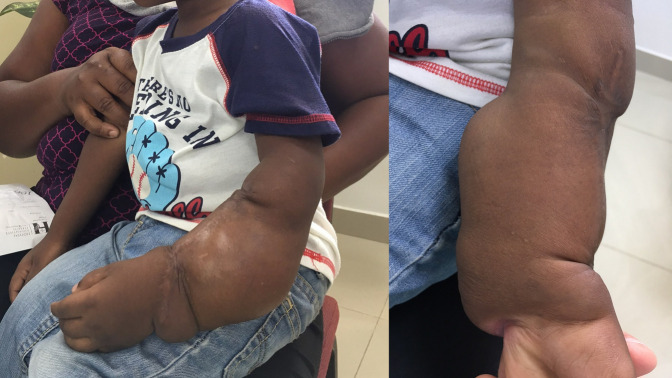
Clinical photographs demonstrating a large tumor involving the left wrist and forearm of a 2-year-old boy. Biopsy resulted in diagnosis of lipofibromatosis and he was treated with resection of the tumor.

A 2-year-old boy presented in Haiti with a mass involving his left forearm that had grown over the previous 2 years after a prior surgical resection at 25 days of age (Figure 7). The resected specimen was never analyzed. The mass was nontender, and the patient had a normal neurovascular examination of the left upper extremity but had increasing difficulty using the arm due to the mass. An incisional biopsy was performed under the guidance of the orthopaedic oncologist and was consistent with lipofibromatosis. Although lipofibromatosis is benign, the mass had progressed substantially over the course of the patient's life. The patient underwent resection of the tumor at 30 months of age. Two years after surgery, the patient is without recurrence and has full function with no deficits.

Although this tumor is benign, the complexity of resection in this case should not be underestimated. There needs to be a thorough understanding of the risk of blood loss, which can be substantial in tumor surgery. In a pediatric patient, minimization of blood loss is essential due to their size and overall blood volume. In this case, blood products were obtained from family members but not necessary. Obtaining donor-directed blood should always be considered if blood loss is a concern and no blood bank is available. There must be the understanding that the patient may have to pay for these services. Meticulous attention should be paid to hemostasis and blood loss by the surgeon with frank discussions with the anesthesiologist before and throughout the surgery. Planned use of a tourniquet with the understanding of time limitations of tourniquet use should be accounted for, and a realistic estimation of the surgery should be understood, erring on a longer procedure. If the location of the tumor prohibits tourniquet use, the surgery should be seriously questioned. The surgeon should treat every case as if blood is not available to minimize losses, and the patient and surgeon should be willing to proceed with an amputation if bleeding is not controlled.

## Surgical Considerations

Limb-salvage surgery is a highly specialized area of orthopaedic surgery, and availability of fluoroscopy, instrumentation, and implants may limit reconstruction options. As tissue banks are unlikely to be available, allograft is usually not an option. Similarly, prosthetic reconstructions may require donation from a company if this is chosen.

Reimplantation of the patient's own bone as an autograft after inactivation by using autoclave,^30,31^ extracorporeal irradiation,^32,33^ alcohol,^34^ and liquid nitrogen^33,35,36^ has been described. Harvest of the autograft, including iliac crest or a free fibula, may be an option. If joint sparing surgery is not possible, fusion of the joint can provide the patient with a functional limb and avoid an amputation. Prolonged periods of non–weight bearing may be needed if there is a large defect and an acute shortening to obtain bony contact is not possible. Distraction osteogenesis and bone transport can also be considerations, but the issues of implant availability, compliance, weight bearing, prognosis, and functional capacity should again be seriously considered. Adaptation to the environment is necessary by the surgeon, and this should be done over a period of time with progressively more complex operations. Surgeons must understand their own limitations in addition to the limitations of the environment and avoid attempting a procedure they do not routinely perform.

## Postoperative Care, Follow-up, and Outcomes

Postoperative care of patients with musculoskeletal tumors is particularly important and should play a part in the choice of treatment undertaken. Follow-up to monitor for recurrence should be performed. A local surgeon should be available and be able to treat complications. If a patient undergoes limb salvage and subsequently requires an amputation, the resultant secondary amputation should ideally not cause a greater functional deficit than the primary amputation would cause. Before enacting a treatment plan, the surgeon must consider if the facility, local surgeons, and the patient and their family have the means and resources to deal with potential complications. If not, proceeding with limb salvage may be more of a disservice to the patient and cause notable harm.

An example of this was illustrated in the case of a patient from rural Kenya who underwent total hip arthroplasty by a nonlocal orthopaedic team and was later diagnosed with a periprosthetic tuberculosis infection.^37^ This complication was unable to be treated by the local healthcare system, and the morbidity of the complication exceeded her presurgical symptoms and limitations. The operating surgeon needs to be willing to make a long-term commitment and have realistic expectations and confidence that the patient will have adequate postoperative care and follow-up. An initiative to develop sustainable orthopaedic care in Tanzania demonstrated the importance of follow-up by implementing year-round coverage by international advisors for the initial 4 to 5 years and emphasizing enhanced local surgical education to enable ultimate transition of surgical capacity to African surgeons.^9^

In our presented cases, limb salvage was possible because of the availability of necessary resources and continuity of care provided by a group of full-time local and international surgeons. These full-time surgeons were capable of providing long-term follow-up and management of any short-term or long-term complications. Moreover, the orthopaedic oncologist remains in contact with the full-time surgeons, is available to discuss treatment of any complications or interventions on an as needed basis, and is willing to travel to the region if needed.

Long-term follow-up should be obtained in patients undergoing both operative and nonoperative care. Complications, treatment modalities, and outcomes should be meticulously recorded to provide feedback as to the successes and failures of the program. All patients had access to cell phones and were contacted routinely to ensure follow-up. Family and neighbor contacts should also be obtained in case the patient loses phone access. Costs of the surgery to the patient and hospital should be evaluated along with donations received to determine economic viability. Table 2 provides an example of a follow-up form that can be used in addition to the information from the patient's chart.

**Table 2:** Follow-up Form

Patient name:											
Medical record number:											
Phone number:											
Family/neighbor contacts:											
Age:											
Sex:											
Diagnosis:											
Date of initial visit:											
Date of surgery:											
Surgeons:											
Anesthesiologist:											
Type of anesthesia:											
Surgery performed:											
Tourniquet time:											
Implants used:											
Extra testing/costs/equipment:											
Disposable equipment used:											
Donations received:											
Overall costs minus donations received:											
Blood loss:											
Was blood donated/by whom:											
Was blood needed:											
Margins status:											
No. of days in the hospital:											
Complications:											
Follow-up schedule/testing required:											
Functional score used/initial score:											
Follow-up (mo):	1.5	3	6	9	12	15	18	21	24	27	30
Recurrence (yes/no)											
Metastasis (yes/no)											
Complications (yes/no)											
Functional score											
Follow-up (mo):	33	36	39	42	45	48	51	54	57	60
Recurrence (yes/no)											
Metastasis (yes/no)											
Complications (yes/no)											
Functional score											
Complications in detail:																						
Comments:											

## Amputations and Prosthetics

In multiple studies from low-income and middle-income countries, cancer is among the top four reasons for amputation.^38,39,40,41^ Basic function is the typical goal for a patient in the resource-limited environment. Unfortunately, the rate of prosthesis fitting after lower extremity amputation can be as low as 24%.^39^ Other estimations report that only 5% to 15% of people in low-income countries have access to assistive devices, with prosthetics being among the most frequently studied.^42,43,44^ Factors limiting the use and success of prosthetics extend beyond availability to the cost to the patient for the prosthetic, lack of proper manufacturing materials, lack of personnel for rehabilitation programs, inadequate public relations campaigns to finance and maintain programs, insufficient knowledge to implement logistical elements, and inadequate practitioner training.^45,46,47^ In rural environments, prosthetics must be affordable, durable, easy to fabricate and repair, culturally acceptable, and able to be used in local conditions which often involves heat, humidity, and manual labor.^43,47,48,49,50^ In the future, three-dimensional printing may make low-cost, custom, simple prosthetic limbs a possibility, but improvements in technology are needed to increase functionality and affordability.^51^ Even those who obtain a prosthesis may be limited by travel costs associated with maintenance.^52^ Although designs and programs have been created to address these needs, there is a lack of outcome data in the literature regarding the use of prosthetics in resource-limited environments.^53^ One study from South Africa reported that only 42% of patients with a prosthesis used it daily and only 30% could walk at least 500 steps.^54^ The authors suggested that these limitations may be due to a lack of rehabilitation, the long time between amputation and prosthetic fitting, or other factors such as the prosthetic component used. Owing to the complexity of challenges related to increasing access to, and success of, prosthetics in resource-limited environments, avoiding amputation in these locations can be even more important than in countries where ample resources exist for prosthetic support.

Even when prosthetic needs are met, amputees face functional problems that can be avoided when limb salvage is possible. In lower extremity amputees, walking speed decreases whereas oxygen consumption increases.^55^ The level of amputation is an important predictor of oxygen consumption, and the metabolic cost increases by 25% to 40% in transtibial amputees and 68% to 100% in transfemoral amputees.^56,57^ Upper extremity amputees lose the ability to perform activities of daily living and have markedly greater combined disability scores than lower extremity amputees.^58^ Upper extremity prosthetics can be cumbersome and lack function, and amputees may use them only to assist with certain activities.^59^

Phantom limb pain and phantom sensations occur frequently after lower and upper extremity amputation, with prevalence in the upper extremity reported to be 51% and 76%, respectively.^60^ Even with treatment modalities, phantom limb pain remains a substantial problem.^61^ When limb salvage is possible, these side effects and challenges can be avoided.

## Summary

Development of partnerships between medical missions and established training programs makes limb salvage for musculoskeletal tumors a greater possibility. A thorough understanding of the facility, anesthesia support, and instrumentation available is vital. The surgeon should travel to the region to perform smaller surgeries and assess outcomes before performing complex reconstructions. Resources vary considerably between countries or even between facilities within the same country. There should be the understanding that advanced imaging, implants, blood products, and allograft are likely unavailable or difficult to obtain. The availability of prosthetics and resources for amputees varies widely, and limb salvage may provide a significant advantage. Established continuity of care, outcome data, and training of local surgeons and staff should be provided. Patients with metastatic disease, likelihood of substantial blood loss, poor health, or with tumors that require treatments that are unavailable in that location are not candidates for limb-salvage surgery. Surgery should only be considered if it is safe, provides more of a benefit than an amputation or palliative care, and is performed with informed consent.
